# Tuberculous meningitis is associated with higher cerebrospinal HIV-1 viral loads compared to other HIV-1-associated meningitides

**DOI:** 10.1371/journal.pone.0192060

**Published:** 2018-02-02

**Authors:** Ikanyeng D. Seipone, Ravesh Singh, Vinod B. Patel, Avashna Singh, Michelle L. Gordon, Daniel M. Muema, Keertan Dheda, Thumbi Ndung’u

**Affiliations:** 1 HIV Pathogenesis Programme, Doris Duke Medical Research Institute, Nelson R. Mandela School of Medicine, University of KwaZulu-Natal, Durban, South Africa; 2 Department of Microbiology, National Health Laboratory Services, KwaZulu-Natal Academic Complex, Inkosi Albert Luthuli Central Hospital, Durban, South Africa; 3 Department of Neurology, Inkosi Albert Luthuli Central Hospital and the Nelson R. Mandela School of Medicine, University of KwaZulu-Natal, Durban, South Africa; 4 Africa Health Research Institute, Durban, South Africa; 5 Kenya Medical Research Institute-Wellcome Trust Research Programme, Centre for Geographic Medicine Research-Coast, Kilifi, Kenya; 6 Lung Infection and Immunity Unit, Division of Pulmonology & Lung Institute, Department of Medicine, University of Cape Town, Cape Town, South Africa; 7 Institute of Infectious Disease and Molecular Medicine, University of Cape Town, Cape Town, South Africa; 8 Ragon Institute of MGH, MIT and Harvard University, Cambridge, Massachusetts, United States of America; 9 Max Planck Institute for Infection Biology, Berlin, Germany; University of Nebraska Medical Center, UNITED STATES

## Abstract

To gain a better understanding of the immunopathogenesis of tuberculous meningitis (TBM) and identify potential diagnostic biomarkers that may discriminate TBM from other HIV-1-associated meningitides, we assessed HIV-1 viral load levels, drug resistance patterns in antiretroviral therapy (ART)-experienced patients with persistent viremia and soluble immunological analytes in peripheral blood and cerebrospinal fluid (CSF) of HIV-1 infected patients with TBM versus other meningitides. One hundred and three matched blood and CSF samples collected from HIV-1 infected patients with TBM or other meningitides presenting at a hospital in Durban, South Africa, from January 2009 to December 2011 were studied. HIV-1 RNA and 28 soluble immunological potential biomarkers were quantified in blood plasma and CSF. Viremic samples were assessed for HIV-1 drug resistance mutations. There were 16 TBM, 46 probable TBM, 35 non-TBM patients, and six unclassifiable patients. TBM and non-TBM patients did not differ in median plasma viral load but TBM patients had significantly higher median CSF viral load than non-TBM participants (p = 0.0005). No major drug resistance mutations were detected in viremic samples. Interleukin (IL)-1β, IL-17, platelet derived growth factor (PDGF)-BB, granulocyte colony stimulating factor (G-CSF) and cathelicidin were significantly elevated in the CNS of TBM participants compared to other patients although these associations were lost after correction for false discovery. Our data suggest that TB co-infection of the CNS is associated with enhanced localized HIV-1 viral replication but none of the evaluated soluble immunological potential biomarkers could reliably distinguish TBM from other HIV-associated meningitides.

## Introduction

HIV-1-associated opportunistic infections of the central nervous system (CNS) pose a major public health problem in resource-limited settings. The most common opportunistic infection in HIV1-infected individuals in sub-Saharan Africa is tuberculosis (TB) [1]. It has been reported that co-infection with HIV-1 and tuberculosis exacerbates both diseases, although the underlying mechanisms remain poorly understood [2]. In particular, the immunological and biological predisposing factors for *Mycobacterium tuberculosis (M*.*tb)* dissemination and reactivation in the CNS of HIV-1 infected people are unclear. Likewise, the impact of tuberculosis in the CNS on HIV-1 replication dynamics and evolution are unknown and data are particularly lacking for sub-Saharan Africa, where HIV-1 is most diverse. Furthermore, rapid and accurate diagnosis of tuberculous meningitis (TBM) is a challenge in developing countries, and morbidity and mortality have increased as a result of HIV/AIDS [3–5]. Therefore, there is an urgent need to better understand the pathogenesis of HIV-1-associated TBM and to identify biomarkers that may point to possible therapeutic interventions or improved diagnosis.

It has been reported that *M*.*tb* enhances HIV replication in alveolar macrophages and peripheral blood T cells through cytokine- and antigen-mediated cellular activation, which in turn leads to higher viral loads and disease progression [6–8]. Although not much is known about the interaction of HIV-1 and TB in the CNS, it has been reported that TBM is associated with higher plasma and CSF viral loads compared to other meningitides [9,10]. The relative concentrations of cytokines and chemokines that modulate HIV replication have been shown to differ between the plasma and CNS compartments [11]. Some cytokines such as interleukin (IL)-1β, IL-6, IL-8, IL-10, interferon (IFN)-γ and tumor necrosis factor (TNF)-α have also been reported to be elevated in the CSF of TBM patients compared to other forms of meningitis [12–14]. It is therefore plausible that cytokines play an integral role in the immunopathogenesis of TBM in HIV-1 co-infected persons and a better understanding for their role could potentially inform therapeutic interventions or help improve the diagnosis and outcome. Another consideration in the clinical management of TBM is the effectiveness of antiretroviral drugs, which are known to lower viremia and improve clinical outcomes [15–17]. However, some of the antiretroviral drugs presently available in sub-Saharan Africa have poor penetration through the blood-brain barrier [18]. This may result in viral persistence, emergence of resistance within the CNS and ultimately neurocognitive impairment [19–21]. Regardless, suppression of plasma viral load is regularly used as an indication of drug effectiveness and as a guide in selecting treatment options [20,22].

In this study, we hypothesised that there are virological and host immunological biomarkers that could distinguish TBM from other meningitis. We investigated HIV-1 viral load levels of between CSF and blood in patients infected with HIV-1 subtype C presenting with tuberculous versus other meningitis and characterized HIV-1 drug resistance profiles in patients with persistent viremia in either compartment. We also compared concentrations of soluble immunological analytes in plasma and CSF amongst TBM and non-TBM patients to identify a specific TBM biomarker profile.

## Methods

### Study participants

**Fig 1:**
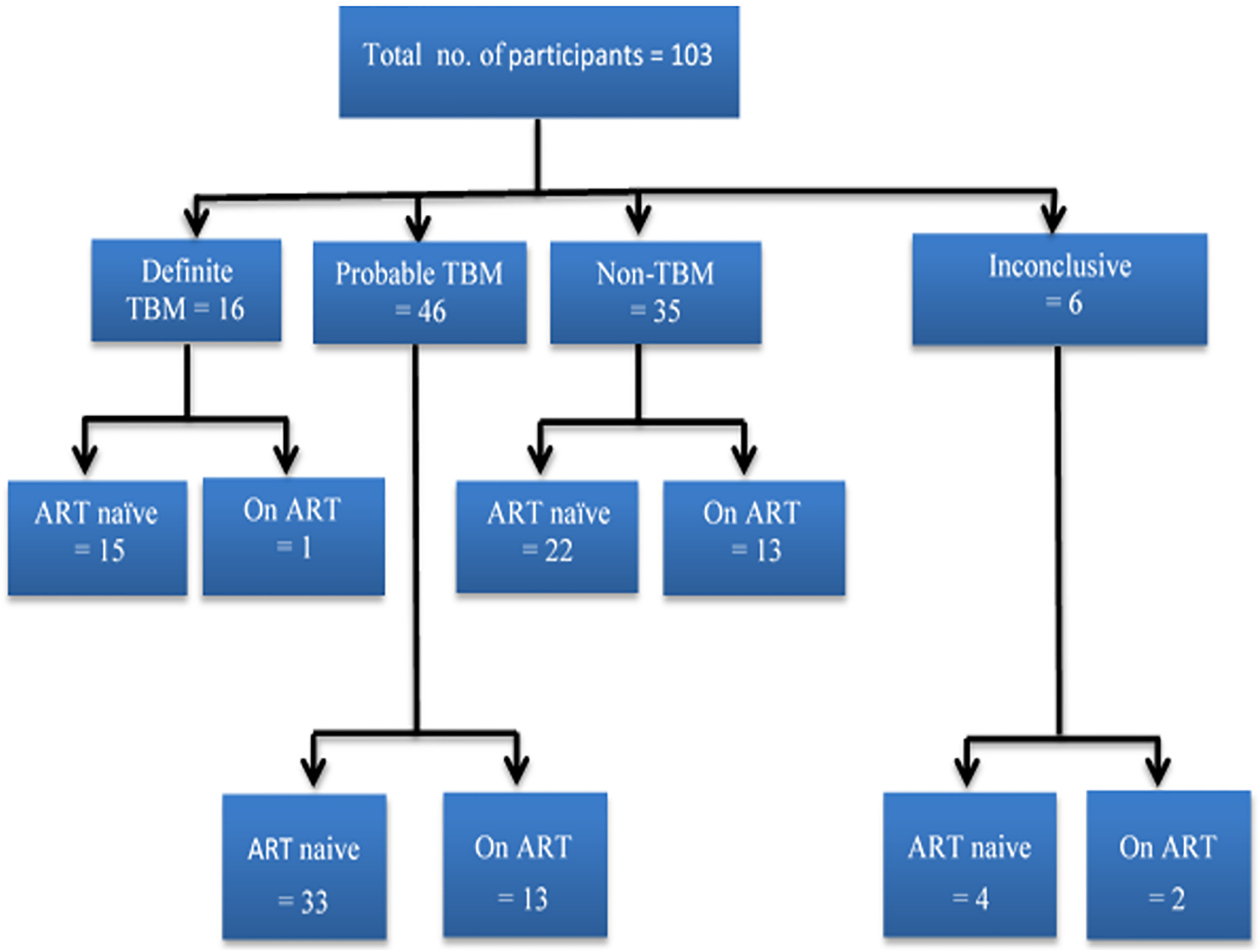
Flow diagram of the study participants and their grouping. Participants with matched plasma and CSF samples were included in the current study. Quantification of viral loads and potential biomarkers were performed on all participants, then viral loads and potential biomarkers comparison analysis were restricted to definite TBM versus non-TBM groups.

Study participants were a subset of individuals with available matched blood and CSF patient samples obtained from patients presenting with meningitides at Inkosi Albert Luthuli Central Hospital in Durban, South Africa as previously described [22,23]. Informed written consent was obtained from all participants, for the patients who were unable provide consent at initial presentation, due to an abnormal mental state, consent was obtained from a first degree relative or from the Head of Department when a lumbar puncture was clinically justified [23,24]. Participants were grouped into three different groups as follows; 1) TBM (either PCR or CSF culture positive for *M*.*tb* (n = 16, with 15 antiretroviral therapy (ART)-naïve), the *M*.*tb* culture and TB PCR (Roche Amplicor, Roche Diagnostics GmbH, Roche Applied Science, Mannheim, Germany) were carried out on fresh CSF obtained by lumber puncture as previously described [23,25]; 2) Probable TBM (clinical features of meningitis, a lumbar puncture (LP) consistent with an aseptic meningitis, negative for other causes of meningitis, and two of the following: a chest X-ray consistent with active pulmonary tuberculosis (PTB), a CT scan consistent with TBM (basal enhancement or hydrocephalus and a response to anti-tuberculous therapy) (n = 46 with 33 ART-naive); and 3) non-TBM (an alternate definite cause for meningitis identified and response to appropriate non-tuberculous therapy) (n = 35 with 22 ART-naive). Detailed description and detection methods for the non-TBM group opportunistic infections are described in previous publications [23,24]. Briefly, the non-TBM group consisted of patients positive for cytomegalovirus (CMV), herpes simplex virus (HSV type 1) varicella zoster virus (VZV) detected by viral PCR (Roche Amplicor), neurosyphilis was detected by fluorescent treponemal antibody (FTA) test and venereal disease research laboratory (VDRL) test if FTA was positive; cysticercal enzyme linked immunosorbent assay (ELISA), and a cryptococcal antigen latex agglutination test (CLAT) were also carried out [23,24]. The ELISPOT assay (T-SPOT.TB; Oxford Immunotec, Oxford, Abingdon, UK) was used to rule out latent TB infection in non-TBM patients as detailed in a previous publication [24]. Six of the patients with suspected meningitis could not be classified as any of the above groups and 4 of these were ART-naïve. Overall, 74 study participants were ART-naïve and 29 were ART-exposed (Fig 1). The participants on ART were all on first line regimen as recommended by the South African Antiretroviral Treatment Guidelines, which includes two nucleoside reverse transcriptase inhibitors (NRTI) and one non-nucleoside reverse transcriptase inhibitor (NNRTI) [26]. Regimen 1A consists of stavudine lamivudine and efavirenz; and regimen 1B is a combination of stavudine, lamivudine and nevirapine. Routine CD4^+^ T-cell counts and clinical chemistry assays performed included CSF glucose, lymphocyte counts and protein levels as previously described [23]. Plasma samples were aliquoted and stored at -80°C and thawed once at use.

### Viral loads

Plasma and CSF HIV-1 viral RNA copies were quantified using the COBAS TaqMan HIV-1 Test, v2.0, with a dynamic detection range of 34 to 10,000,000 copies/ml (Roche Molecular Systems, Inc. Branchburg, NJ, USA). The plasma and CSF samples of each patient were assayed in parallel to prevent inter-assay variations.

### Genotypic resistance testing

Of the 29 patients on ART, four patients had persistent viremia in both plasma and CSF, three patients had persistent plasma viremia only and one patient had persistent viremia in the CSF only. Persistent viremia was defined as being on treatment but with HIV-1 viral loads ≥400 copies/ml. An in-house HIV-1 drug resistance assay was performed on the samples with persistent viremia in the CSF, plasma or both. Briefly, viral RNA was extracted from both CSF and plasma using the QIAamp Viral RNA Mini Kit (Qiagen, Hilden, Germany), followed by cDNA synthesis and amplification of the *pol* gene as previously described [27]. PCR products were then purified using the Qiagen PCR purification kit (Qiagen) and sequenced using the Sanger method (3130XL Genetic Analyzer; Applied Biosystems, Foster City, CA, USA). Sequences were assembled and manually edited using Sequencher software version 5.0 (Gene Codes Corporation, Ann Arbor, MI, USA). Clustal X version 2.0 [28] was used to align sequences, followed by manual editing using BioEdit version 7.2.2 [29]. To ensure no cross-contamination between samples, neighbour joining phylogenetic trees were drawn and viewed using the Geneious software suite (Biomatters Ltd., Auckland, New Zealand). Sequences were submitted to the Stanford Drug Resistance Database, for identification of drug resistance mutations (DRMs) (http://hivdb.stanford.edu).

### Potential biomarkers measurement

Analyte levels of paired plasma and CSF samples were measured using the Bio-Plex Pro^™^ 27-plex Luminex kit (BioRad Laboratories Inc, Hercules, California, USA) according to the manufacturer’s instructions, with modifications as detailed hereafter. The master standard stock was diluted 10X, following the normal four-fold dilution of the standard as per manufacturer’s instructions. The panel included: pro-inflammatory cytokines; IL-1β, IL-2, IL-7, IL-9, IL-12, IL-17, IFN-γ and TNF-α; anti-inflammatory cytokines interleukin 1 receptor antagonist (IL-1ra), IL-4, IL-5, IL-6, IL-10, IL-13, IL-15 and tumor growth factor (TGF)-β; chemo-attractants IL-8, monocyte chemotactic protein (MCP)-1, macrophage inflammatory protein (MIP)-1α, MIP-1β and IFNγ–induced protein (IP)-10, regulated upon activation normal T-cell expressed and activated (RANTES) and eotaxin; growth factors granulocyte macrophage colony-stimulating factor (GM-CSF), granulocyte colony-stimulating factor (G-CSF), vascular endothelial growth factor (VEGF), platelet derived growth factor (PDGF)-BB and fibroblast growth factor (FGF)-basic. Results were read on the BioPlex MAGPIX^™^ reader and analyzed on the Bio-Plex Manager software 6.1 (BioRad). The levels of the antimicrobial peptide cathelicidin LL-37 (CAMP) were measured using an enzyme-linked immunosorbent assay (ELISA) kit (USCN Life Science Inc., Wuhan, China). Cathelicidin LL-37 is an antimicrobial peptide produced by neutrophils and macrophages after activation by bacteria or vitamin D [30].

### Statistical analysis

Descriptive statistics are presented as medians (with interquartile ranges) or means (with standard errors). Comparison of groups for viral loads and analytes levels were done using the Mann-Whitney test for non-parametric data in GraphPad Prism software (version 5.01; GraphPad). Spearman rank correlation was used to determine relationships between continuous variables. To elucidate whether there is a specific biomarker profile for TBM, unsupervised hierarchical clustering (UHC) and heat maps were generated in Morpheus (https://software.broadinstitute.org/morpheus/) using 1- (spearman rank correlation) as a measure of dissimilarity. Principal component analysis (PCA) was also performed to further assess the biomarker profile for TBM. In multivariable analyses to assess the prediction of TBM by each analyte after adjusting for other possibly confounding analytes, a logistic regression model incorporating the analytes that were significantly different in the initial Mann-Whitney test between TBM and non-TBM groups was used. All analyses were done using GraphPad Prism software (version 5.01; GraphPad) and STATA version 13. *P*<0.05 were considered significant.

### Ethical approval

The research study was approved by the Biomedical Research Ethics Committee of the University of KwaZulu-Natal (Reference: E325/05).

## Results

There was a total of 103 patients (16 TBM, 46 probable TBM, 35 non-TBM and 6 patients could not be classified into any of these groups) and out of these 74 were ART-naïve with the remaining 29 on ART. The median age of the participants was 32 with an interquartile range [IQR] of 28–36 and 33 [28–38] years respectively, with the majority being male for both the ART-naïve (60%) and those on ART (76%) (S1 Table). Forty percent of ART-naïve TBM (n = 15) participants and 86% of the ART-naïve non-TBM (n = 22) participants were male (p = 0.005) (Table 1). The median age of the ART-naïve (32 [IQR, 29–35]) participants did not differ from the non-TBM ART-naïve (34 [30–37]) participants (p = 0.438) (Table 1). There was only one TBM participant on ART hence comparison of the demographics could not be done between the TBM and non-TBM treated participants (Table 1).

**Table 1:** Comparison of demographic and clinical characteristics of TBM and non-TBM study participants.

Category	Characteristics	Definite TBM	Non-TBM	P value
**ART-NAIVE**	No of patients	15	22	
	Sex % male	40%	86%	**0.005**^#^
	Median Age (IQR) in years	32 (29–35)	34 (30–37)	0.438*
	Median CD4 counts (IQR) cells/μl	78 (41–138)	161 (45–286)	0.050*
	Median Lymphocytes (IQR) cells/μl	5.0 (1.0–11)	2.0 (0.3–4.2)	0.031*
	Median Proteins (IQR) g/l	2.0 (1.9–2.7)	1.0 (0.7–1.7)	0.003*
	Median Glucose-CSF (IQR) nnonnmol/l nmol/l nmol/l nmol/l	1.3 (1.0–1.8)	2.3 (1.7–2.7)	0.019*
	% pleocytosis	47%	18%	0.080^#^
	% with Glucose-CSF < 2.2 nmol/l	87%	67%	0.262^#^
	% with Protein-CSF > 0.46g/l	100%	91%	0.505^#^
**ON ART**	No of patients	1	13	
	Sex % male	100%	31%	N/D
	Median Age (IQR) in years	33 (N/A)	36 (33–45)	N/D
	Median CD4 counts (IQR) cells/ μl	81 (N/A)	130 (76–336)	N/D
	Median Lymphocytes (IQR) cells/ μl	2.9 (N/A)	2.1 (0.8–5.0)	N/D
	Median Proteins (IQR) g/l	3.3 (N/A)	0.9 (0.6–2.7)	N/D
	Median Glucose-CSF (IQR) nmol/l	0.6 (N/A)	1.7 (0.9–3.4)	N/D
	% pleocytosis	0%	7.70%	N/D
	% with Glucose-CSF < 2.2 nmol/l	0%	62%	N/D
	% with Protein-CSF > 0.46g/l	100%	92%	N/D

N/D: Not determined because there was only one TBM patient on ART. N/A: Not applicable. IQR: Interquartile range. Significant differences between the groups are shown in bold P values

*Mann-Whitney test

^#^Fisher’s exact test

**Fig 2:**
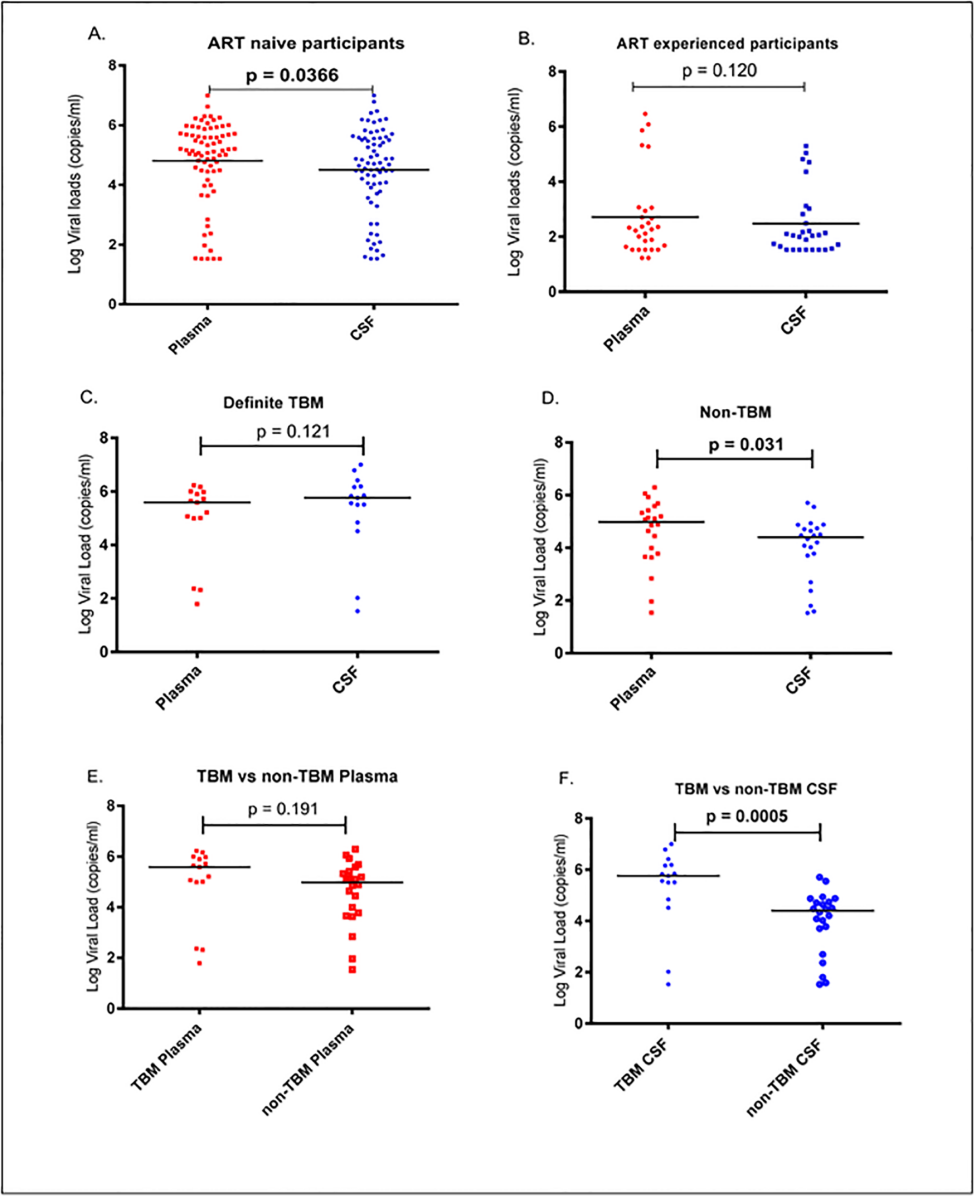
HIV-1 viral loads in plasma and cerebrospinal fluid (CSF). (A) Plasma viral loads are significantly higher than CSF viral loads in ART-naïve participants. (B) For ART-experienced participants with meningitis, there was no significant difference in HIV-1 viral load levels when comparing plasma versus CSF. (C) HIV-1 viral loads in the CSF versus plasma of ART-naïve patients with tuberculous meningitis (TBM), showing no significant differences between the two compartments. (D) ART-naïve non-TBM patients had significantly higher plasma compared to CSF viral loads. (E) Plasma viral loads of ART-naïve patients with TBM versus other meningitides are not significantly different. (F) Significantly higher HIV-1 viral loads in CSF of ART-naïve TBM patients compared to those with other meningitides. P-values are shown for each comparison and are bolded if the differences are statistically significant.

HIV-1 target cells and the immunological milieu that facilitate viral replication may differ between peripheral blood and the CNS resulting in discordant levels of the virus in the two compartments within an individual. Among ART-naïve individuals, comparison of plasma and CSF viral loads showed that the median plasma viral load was 5.14 log_10_ copies/ml (interquartile range [IQR, 4.46–5.72]), significantly higher than the median CSF viral load of 4.70 log_10_ copies/ml, [3.88–5.57] (p = 0.0366) (Fig 2A). There were no significant differences between CSF and plasma viral loads for the 29 patients on ART (Fig 2B), consistent with suppression of viremia by ART in the two compartments. Some participants had detectable viremia above 400 copies/ml in either compartment suggesting non-adherence, emergence of drug resistance or lack of drug penetration into the CNS.

HIV-1 viral load dynamics in the CNS in the context of opportunistic infections is poorly understood. We investigated whether tuberculous meningitis is associated with differential viral replication between the blood and CNS compartments. This analysis was first performed on ART-naïve patients by comparing definite TBM patients’ CSF and plasma viral loads. TBM patients showed no significant viral load differences between the blood (median 5.59 log_10_ copies/ml [IQR, 5.00–5.98]) and CSF (5.76 log_10_ copies/ml [IQR, 4.85–6.19]) compartments (p = 0.240) (Fig 2C). For patients with an alternate cause of meningitis (i.e. the non-TBM group), plasma viremia (median 4.98 log_10_ copies/ml [IQR, 3.76–5.47]) was significantly higher than CSF viremia (4.40 log_10_ copies/ml [IQR, 3.46–4.78]) (p = 0.031) (Fig 2D).

We next assessed whether TBM was associated with elevated CSF HIV-1 viremia as has previously been reported among ART-naïve patients [9]. Among ART-naïve patients, median plasma viral loads did not differ significantly between the TBM (5.59 log_10_ copies/ml [IQR, 5.00–5.98]) and non-TBM (4.98 log_10_ copies/ml ([IQR, 3.76–5.47]) groups (p = 0.240) (Fig 2E). Remarkably however, in TBM patients, the median CSF viral load (5.76 log_10_ copies/ml, [IQR, 4.85–6.19]) was significantly higher than the non-TBM CSF viral load (4.40 log_10_ copies/ml [IQR 3.46–4.78]) (p = 0.0005) (Fig 2F). Overall, these data indicate that TBM is associated with higher HIV-1 replication in the CNS but not in plasma although we cannot distinguish between cause and effect due to the cross-sectional design nature of our study.

CNS inflammation may contribute to viral replication in the CNS irrespective of etiology. Detailed findings from our study cohort on indicators of CNS pathology have been presented elsewhere [12]. Here, we first explored the relationship between pleocytosis (a common marker of inflammation and of disruption of the blood brain barrier, defined as >5 cells/mm^3^ in the CNS [31] with ART use and meningitis etiology. The TBM group had the highest median number of lymphocytes (5.0 cells/μl, [IQR, 1.0–11.0]) compared to the non-TBM group (2.0 cells/μl, [IQR, 0.3–4.0]) (p = 0.031). The TBM group also had the highest percentage of patients with lymphocytic pleocytosis (47%), however, it was not statistically different from the non-TBM group (18%) (p = 0.080) (Table 1). The median number of CSF lymphocytes was within the normal range for the non-TBM group (2.0 cells/μl, [IQR, 0.3–4.0]) (Table 1). Other commonly used clinical markers of CNS inflammation are CSF protein and glucose concentrations [32]. Glucose levels of >2.2 mmol/*l* in the CNS are regarded as normal, with lower levels taken as an indication of bacterial infection. Comparison within ART-naïve participants showed that the definite TBM group had abnormal median glucose levels and the median glucose levels (1.3 nmo/l, [IQR, 1.0–1.8]) were significantly lower than the non-TBM group (2.3 nmol/l [IQR, 1.7–2.7]) (p = 0.019). All the TBM group patients (100%) had abnormal protein levels, above the normal cut-off concentration of 0.46 g/l (Table 1). There was significant positive correlation between the HIV-1 CSF viremia and the number of CSF lymphocytes for both TBM patients (r = 0.518, p = 0.0240) and non-TBM patients (r = 0.410, p = 0.0292). There was no correlation between CSF glucose, protein, or CD4 counts and CSF viremia (S2 Table).

Among patients on antiretroviral therapy, viral load discordance between peripheral blood and the CNS has occasionally been reported and may indicate ongoing compartmentalized virus replication [19,21,33–35]. We sequenced the *pol* gene to assess for drug resistance-associated mutations in patients with persistent viremia in peripheral blood or the CSF. Four patients (A83, A102, A128 and A138) had persistent viremia in both plasma and CSF. Patient A83 had been on ART for 7 months, A102 for 24 months, whereas treatment duration was unknown for A128 and A138. None of these patients showed major DRMs, however patient A102 had the T74S Protease Inhibitor (PI)-accessory mutation in both compartments. Patients X219, A149 and X225 had persistent viremia in plasma only and had been on treatment for 1, 28 and 24 months respectively. No DRMs or polymorphisms were present in X219. Patient A149 harboured the T74S PI-accessory mutation and X225 had both the A98G NNRTI-non-polymorphic accessory mutation and the T74S PI-accessory mutation. Only patient A107 had persistent viremia in the CNS only, and this patient had been on treatment for two months. Patient A107 had no major drug resistance mutations but had the E138A NNRTI-polymorphic mutation. Overall, resistance profiling of the patients only showed polymorphic and accessory mutations on 4 patients (Table 2).

**Table 2:** Demographic, clinical characteristics and summary of drug resistance testing profiles of patients with persistent viremia treated with first-line antiretroviral therapy.

Participant ID	Sex	Age (years)	Source Compartment	CD4 count cells/μl	Plasma and CSF viral loads (copies/ml)	Treatment regimen	Treatment duration (months)	NRTI (Y/N)	NNRTI (Y/N)	PI (Y/N)
A83	F	36	Plasma	245	724761	1B	7	N	N	N
			CSF		65465			N	N	N
A102	F	25	Plasma	159	2912258	1A	24	N	N	Y (T74S)
			CSF		23088			N	N	Y (T74S)
A107	F	27	CSF	234	1316	1A	2	Y (E138A)	N	N
A128	F	23	Plasma	28	211518	1B	no info	N	N	N
			CSF		51013			N	N	N
A138	F	37	Plasma	97	1143	1B	no info	N	N	N
			CSF		670			N	N	N
A149	F	29	Plasma	95	871	1A	28	N	N	Y (T74S)
X219	M	19	Plasma	62	470	1A	1	N	N	N
X225	F	36	Plasma	ND	1180	1A	24	N	Y (A98G)	Y (T74S)

Regimen 1A: stavudine lamivudine, efavirenz; regimen 1B: stavudine, lamivudine, nevirapine; NRTI, nucleoside reverse transcriptase inhibitor; NNRTI, non-nucleoside reverse-transcriptase inhibitor; PI, protease inhibitor; N, no; Y, yes; no info, no information available; ND, not done.

Identification of immunological changes specific to particular pathogens could present an opportunity for better understanding of pathogenesis and therapeutic interventions or improved diagnosis. Therefore, we first compared analyte levels between compartments and thereafter explored for potential plasma and CSF immunological biomarkers that may distinguish between TBM and non-TBM meningitis. Comparisons of median concentrations of plasma and CSF analytes between TBM and non-TBM patients were performed as well as unsupervised hierarchical clustering (UHC) and principal component analysis (PCA) to elucidate whether there is a specific biomarker expression profile for TBM in HIV-1 co-infected patients.

**Fig 3:**
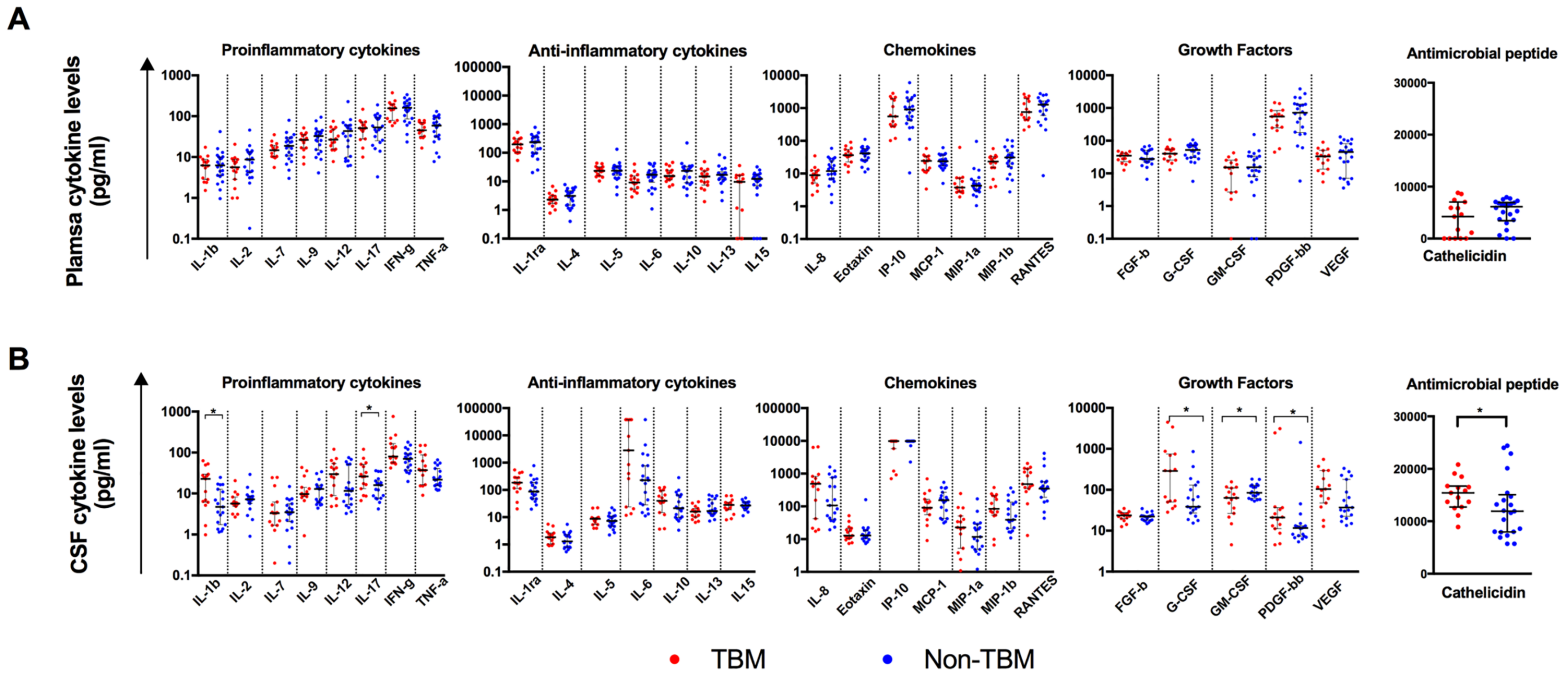
Plasma and CSF biomarker levels. Comparison of potential biomarker levels in (A) plasma and (B) CSF of ART-naïve patients with and without TBM. * p < 0.05, statistical test, Mann Whitney test.

Comparison of plasma and CSF analyte levels of all ART-naïve participants showed that the following were significantly higher in the CSF than plasma; IL-β, IL-6, IL-8, IL-10, IL-15, G-CSF, GM-CSF, IP-10, MCP-1, MIP-1α, MIP-1β, VEGF and cathelicidin (Table 3) however after Bonferroni adjustment for multiple comparison IL-1β was not significant. In contrast, the levels of IL-1ra, IL-2, IL-4, IL-5, IL-7, IL-9, IL-12, IL-17, eotaxin, FGF-b, IFN-γ, PDFG-bb, RANTES and TNF-α were significantly higher in plasma compared to CSF, IL-1ra, IL-2 lost significance after Bonferroni adjustment for multiple comparison. We next compared analyte levels amongst groups (for ART-naïve patients only). There was no significant difference between TBM and non-TBM patients in any of the plasma analyte levels (Fig 3A). The pro-inflammatory cytokines IL-1β and IL-17 were significantly higher in the CSF of TBM patients compared to non-TBM patients (p = 0.0152 and p = 0.0461, respectively). The growth factors PGDF-BB and G-CSF were also higher in CSF of TBM patients compared to non-TBM patients (p = 0.0461 and p = 0.0242 respectively). One growth factor, GM-CSF was significantly higher in non-TBM than TBM patients (p = 0.0407) (Fig 3B). However, upon Bonferroni adjustment for multiple comparisons, none of the analytes were significantly different between the groups. Cathelicidin was significantly elevated in the CSF of TBM patients compared to non-TBM patients (p = 0.0449) (Fig 3B). We then used a multivariable logistic regression analyses to determine the predictive ability of IL-1β, IL-17, PDGF-bb, G-CSF and cathelicidin on TBM after adjusting for each one of these analytes. There were no significant predictions of TBM by any of the analytes. However, for every nanogram increase in CSF levels of cathelicidin, there was a trend whereby the odds of having TBM increased by a factor of 1.376 [95% confidence intervals (0.996–1.900) p = 0.053] (S3 Table).

**Table 3:** Comparison of plasma and CSF potential biomarker levels for all ART-naïve assayed participants.

Biomarker	Plasma	CSF	P-value	Adjusted P-value
IL-1β	7.260 (3.825–11.71)	9.230 (3.365–24.78)	0.0122	0.3294
IL-1ra	237.7 (134.2–363.0)	158.9 (69.47–284.2)	0.0099	0.2673
IL-2	8.830 (4.050–12.65)	6.780 (4.880–9.385)	0.0180	0.486
IL-4	3.380 (2.025–4.920)	1.740 (1.060–2.435)	<0.0001	<**0.0027**
IL-5	24.98 (17.91–34.76)	7.720 (4.880–11.16)	<0.0001	<**0.0027**
IL-6	17.68 (9.075–33.21)	667.8 (60.35–10083)	<0.0001	<**0.0027**
IL-7	18.53 (11.83–26.35)	3.600 (2.275–6.775)	<0.0001	<**0.0027**
IL-8	13.53 (7.895–22.78)	355.2 (62.30–924.9)	<0.0001	<**0.0027**
IL-9	34.57 (21.23–40.60)	11.30 (8.095–16.18)	<0.0001	<**0.0027**
IL-10	21.64 (12.79–33.05)	37.54 (12.17–82.56)	<0.0001	<**0.0027**
IL-12	42.20 (19.27–56.25)	14.91 (7.260–54.12)	0.0107	0.2889
IL-13	17.37 (10.16–25.62)	16.74 (11.09–26.71)	0.3904	>0.9999
IL-15	13.00 (6.540–19.68)	28.40 (21.82–41.66)	<0.0001	<**0.0027**
IL-17	59.92 (32.35–90.65)	18.62 (12.10–30.50)	<0.0001	<**0.0027**
Eotaxin	48.93 (29.61–63.51)	14.38 (11.19–20.01)	<0.0001	<**0.0027**
FGF basic	47.41 (34.75–108.9)	27.34 (22.64–57.73)	<0.0001	<**0.0027**
G-CSF	58.97 (33.13–74.18)	99.76 (41.59–385.4)	<0.0001	<**0.0027**
GM-CSF	20.49 (8.390–36.40)	88.93 (53.86–120.2)	<0.0001	<**0.0027**
IFN-γ	187.0 (110.0–246.9)	82.89 (55.55–152.8)	<0.0001	<**0.0027**
IP-10	902.0 (477.2–1793)	9800 (9800–9800)	<0.0001	<**0.0027**
MCP-1	24.65 (16.99–38.09)	136.6 (64.25–237.8)	<0.0001	<**0.0027**
MIP-1α	4.820 (3.300–7.445)	21.93 (6.930–43.83)	<0.0001	<**0.0027**
PDGF-bb	774.1 (314.3–1293)	14.81 (8.395–35.29)	<0.0001	<**0.0027**
MIP-1β	35.23 (20.81–51.58)	84.08 (34.89–179.5)	<0.0001	<**0.0027**
RANTES	1234 (676.5–1672)	482.3 (229.0–1048)	<0.0001	<**0.0027**
TNF-α	63.63 (30.85–87.48)	36.69 (16.90–60.31)	0.0052	0.1404
VEGF	41.98 (23.04–62.44)	64.34 (24.25–200.4)	<0.0001	<**0.0027**
Cathelicidin	5928.9 (3281.9–7162.7)	12888 (8444.8–15499	<0.0001	-

Statistical test: Wilcoxon matched pairs signed rank test. Adjusted P values were determined by Bonferroni correction. Significant adjusted results are shown with P values in bold

**Fig 4:**
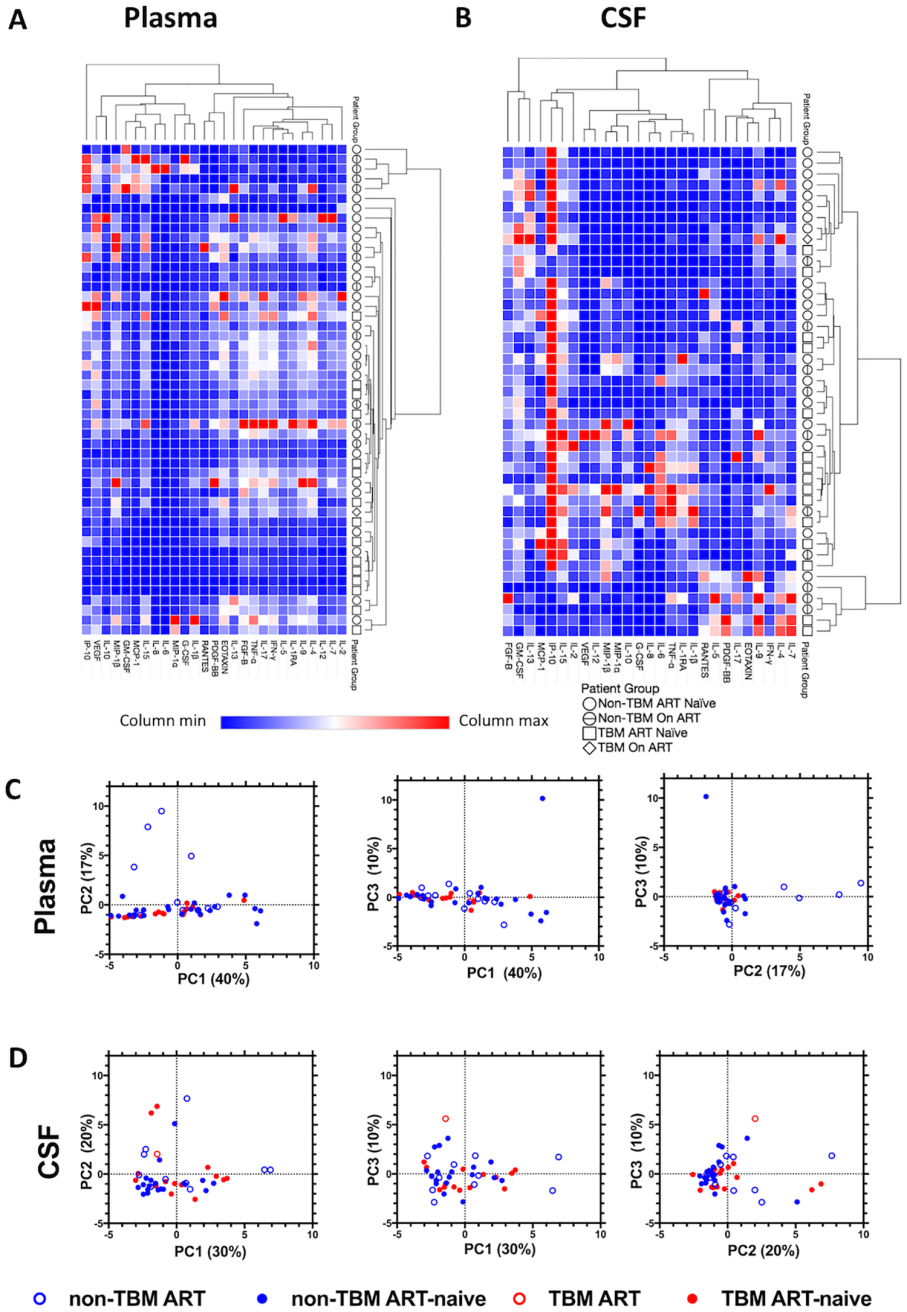
Clustering patterns of potential biomarkers according to participant clinical status (TBM, non-TBM, ART-naïve and ART-experienced). Hierarchical clustering heat map of potential biomarker profiles for (A) plasma analytes and (B) CSF analytes of patients with and without TBM (on ART and not on ART). Blue represents low concentration of biomarker below the median and red depicts high concentration of biomarker above the median value. (C) PCA plots of biomarkers in the plasma and (D) CSF of TBM and non-TBM patients. The % of variance explained by each PC is displayed on the respective axis.

Unsupervised hierarchical clustering on plasma analytes did not reveal any common profile within the TBM or non-TMB groups (Fig 4A). In the unsupervised hierarchical clustering of CSF analytes, the samples formed 3 major clusters of 12, 27 and 6 patients each. However, there were no significant preferential distributions of TBM or non-TBM patients to the clusters, even when the analysis was limited to the ART-naïve patients in the larger clusters of 12 and 27 patients (Fishers exact test, p = 0.13) (Fig 4B). In further assessment using principal component analyses (PCA), there was no evidence of differential clustering of patient groups when the PCA analyses were done on either plasma or CSF analytes, suggesting lack of a strong biomarker signature that can definitively differentiate TBM patients from non-TBM patients (Fig 4C and 4D).

## Discussion

TBM is common but difficult to diagnose definitively in HIV-infected individuals. The true incidence of TBM is not known due to lack of diagnostic tools; however, Patel *et al*., (2013) reported that in the KwaZulu-Natal province of South Africa, approximately 10% of the TB cases are TBM [36]. There is limited information on HIV-1 replication dynamics in the CNS in TB co-infected individuals and the factors that may facilitate viral replication in this compartment are largely unknown especially for subtype C that dominates in sub-Saharan Africa. Our study demonstrated that CSF HIV-1 viremia is higher in TBM versus non-TBM patients, with no significant difference between the plasma viral loads of the two groups. Non-TBM patients had higher plasma compared to CSF viremia, whereas no significant differences between the compartments were noted for the TBM group (Fig 2). Several studies have reported lower levels of HIV-1 in the CSF compared to matched plasma, primarily in patients without opportunistic infections [37–39] and this is consistent with findings among the non-TBM patients in our cohort. Our findings are consistent with previous studies showing that TBM is associated with higher levels of HIV-1 RNA in the CSF compared to other meningitides [9,10]. These findings suggest that TB co-infection enhances HIV-1 replication in the CNS, consistent with studies demonstrating that TB antigens and microbial products enhance HIV replication [40,41]. However, it is also possible that enhanced HIV replication in the CNS predisposes to new infection or reactivation of tuberculosis in this compartment.

The TBM group had higher median number of lymphocytes than the non-TBM group, indicative of higher CNS inflammation in TBM. The TBM group also had the highest percentage of patients with abnormal glucose and lowest CD4 T-cell counts (Table 1). Among the TBM patients, the only clinical marker of CNS inflammation that correlated positively with CSF viral loads was lymphocyte count (S2 Table). Similar to our study, it was previously reported that TBM was characterized by lymphocytic pleocytosis, which was more severe than in other forms of HIV-1-associated meningitis, and that the number of infiltrating lymphocytes correlated positively with HIV-1 viremia [9]. This may indicate that co-infection with TB enhances the inflammation in the CNS more than other meningitides or high inflammation caused by HIV-1 may be leading to TB co-infection. Overall, our data suggest that in TBM there may be more target cells for intrathecal HIV replication and enhanced viral trafficking to the CNS [31].

Discordant plasma and CSF viral loads in persons receiving ART have previously been reported and may indicate low drug penetrance and emergence of drug resistant variants, which may in turn lead to enhanced virus replication and associated neurological deterioration [20,21,37,38]. In this study, there was no evidence of major DRMs in patients with persistent viremia in either compartment, suggesting that treatment failure may be due to sub-optimal drug adherence. One patient (A107) had persistent viremia in the CNS and not in plasma. This patient however had only been on treatment for two months, so it may indicate slower penetration of drugs into the CNS resulting in delayed virus suppression kinetics.

The diagnosis of TBM in the CNS poses a problem in developing countries with high HIV/TB co-infection burden. TB culture is commonly used, but this has low sensitivity and takes weeks before diagnosis [42]. Delay in diagnosis can lead to poor prognosis. Discovering TBM-specific biomarkers in HIV-infected individuals is crucial for efficient clinical management of the infection. Comparison of TBM and non-TBM cases identified CSF immunological analytes, namely IL-1β, IL-17, PDGF-BB, G-CSF and cathelicidin that were significantly elevated in the TBM compared to non-TBM patients. Although our cohort was small, and these analytes lost statistical significance after correction for multiple comparisons, and PCA could not identify a specific TBM specific biomarker profile, these findings may point to a potential role of the pro-inflammatory cytokines (IL-1β and IL-17), growth factors (PDGF-BB and G-CSF) and the antimicrobial peptide cathelicidin in the immunopathogenesis of TBM and to their utility as potential diagnostic biomarkers. Pro-inflammatory cytokines IL-17 and IL-1β play a crucial role in bacterial infections. IL-17 is known to induce many inflammatory cytokines, recruits neutrophils against extracellular bacteria and induces antimicrobial peptides in macrophages. Low levels of this cytokine have been associated with impaired immunity to bacterial infections [43,44]. IL-1β is one of the few cytokines produced by microglia cells in the CNS and it induces activation and proliferation of astrocytes. This cytokine has been implicated in HAD and has been shown to be highly expressed in patients with HAD, and increased production of IL-1β following monocyte activation with toll-like receptor (TLR) ligands has been associated with reduced odds of tuberculosis recurrence [45,46]. The growth factors PDGF and G-CSF are produced by macrophages and endothelial cells. PDGF regulates cell growth and division, blood vessel formation, and it can also be produced by platelets. G-CSF is a glycoprotein that stimulates bone marrow to produce granulocytes and stem cells. This cytokine also stimulates proliferation, differentiation and function of neutrophils [47]. Cathelicidin LL-37 is an antimicrobial peptide produced by neutrophils and macrophages after activation by bacteria or vitamin D [30]. Our data on cathelicidin are consistent with findings from children with TBM who had significantly elevated CSF cathelicidin levels in the TBM compared to non-TBM patients [30], but there are other notable differences for other potential biomarkers analysed, perhaps a reflection of differences between adults and children in disease immunopathogenesis. Nevertheless, our data highlight the potential for CSF biomarkers to distinguish between TBM and other HIV-1-associated meningitides in adults.

## Conclusion

TB co-infection of the CNS is associated with enhanced HIV-1 replication and disruption of the BBB, characterized by lymphocytic CNS inflammation. In this study cohort, there was no evidence of compartment-specific viral escape from antiretroviral therapy. Our study suggests that in TBM, the CNS may serve as a compartment for enhanced HIV replication and may therefore serve as a reservoir. Our study failed to identify soluble immunological biomarkers that could reliably distinguish between TBM from other HIV-associated meningitides although a few, including IL-1β, IL-17, PDGF-BB, G-CSF and cathelicidin may require further evaluation in future studies to improve the understanding of the immunopathogenesis or improved diagnosis of HIV-1-associated adult TBM.

## Supporting information

S1 Table Demographic and clinical characteristics of study participants. (DOCX)

S2 Table Analysis of correlation between viral loads with CD4 counts and markers of inflammation (CSF lymphocytes, proteins, and glucose). (DOCX)

S3 Table The predicted odds of having TB meningitis with variation in levels of biomarkers that were significantly different between TBM and non-TBM groups. (DOCX)
