# Supplementary material for: Tuberculous meningitis is associated with higher cerebrospinal HIV-1 viral loads compared to other HIV-1-associated meningitides
Source: PLoS One. 2018 Feb 2;13(2):e0192060. doi: 10.1371/journal.pone.0192060 (PMC5796705; doi:10.1371/journal.pone.0192060)
Supplement: S1 Table — (DOCX) [file pone.0192060.s001.docx]

**S1 table. Demographic and clinical characteristics of study participants**

| **Category** | **Characteristics** | **All Patients** | **Definite TBM** | **Non-TBM** | **Probable TBM** | **Inconclusive** |
| --- | --- | --- | --- | --- | --- | --- |
|  |  |  |  |  |  |  |
| **ART-NAIVE** | No of patients | 74 | 15 | 22 | 33 | 4 |
|  | Sex % male | 60% | 40% | 86% | 42% | 25% |
|  | Median Age (IQR) in years | 32 (28-36) | 32 (29-35) | 34 (30-37) | 30 (26-36) | 30 (23-34) |
|  | % pleocytosis | 36% | 47% | 18% | 45% | 25% |
|  | % with Glucose-CSF < 2.2 nmol/l | 61% | 87% | 67% | 41% | 25% |
|  | % with Protein-CSF > 0.46g/l | 97% | 100% | 91% | 100% | 100% |
|  | Median CD4 counts (IQR) cells/μl | 161 (60-287) | 78 (41-138) | 161 (45-286) | 239 (122-437) | 116 (46-197) |
|  | Median Lymphocytes (IQR) cells/μl | 3.0 (0.8-7.0) | 4.6 (1.3-10.7) | 1.6 (0.3-4.2) | 4.9 (1.6-10.6) | 2.2 (0.7-4.0) |
|  | Median Proteins (IQR) g/l | 1.8 (1.0-2.6) | 2.0 (1.9-2.7) | 1.0 (0.7-1.7) | 1.95 (1.4 -2.5) | 1.0 (0.8-1.4) |
|  | Median Glucose-CSF (IQR) nmol/l | 1.9 (1.4-2.5) | 1.3 (1.0-1.8) | 2.3 (1.7-2.7) | 1.9 (1.5-2.3) | 2.7 (2.2-3.6) |
|  |  |  |  |  |  |  |
| **ON ART** | No of patients | 29 | 1 | 13 | 13 | 2 |
|  | Sex % male | 76% | 100% | 31% | 7.7% | 50% |
|  | Median Age (IQR) in years | 33 (28-38) | 33 (N/A) | 36 (33-45) | 29 (25-37) | 27 (23-31) |
|  | % pleocytosis | 14% | 0% | 7.7% | 23% | 0% |
|  | % with Glucose-CSF <2.2 nmol/l | 62% | 0% | 62% | 61% | 50% |
|  | % with Protein-CSF > 0.46g/l | 97% | 100% | 92% | 100% | 100% |
|  | Median CD4 counts (IQR) cells/μl | 166 (85-266) | 81 (N/A) | 130 (76-336) | 190 (104 – 240) | 150 (29-270) |
|  | Median Lymphocytes (IQR) cells/μl | 1.2 (0.5-2.7) | 2.9 (N/A) | 2.1 (0.8-5.0) | 1.0 (0.2 -1.4) | 2.0 (1.9 -2.2) |
|  | Median Proteins (IQR) g/l | 1.2 (0.8-2.2) | 3.29 (N/A) | 0.9 (0.6-2.7) | 1.2 (1.0 – 1.9) | 2.1 (1.0-3.1) |
|  | Median Glucose-CSF (IQR) nmol/l | 1.7 (1.2-2.8) | 0.6 (N/A) | 1.7 (0.9-3.4) | 1.8 (1.4 – 2.8) | 2.5 (1.0-4.0) |
|  |  |  |  |  |  |  |

IQR: Interquartile range

N/A: Not applicable
