# Supplementary material for: Tuberculous meningitis is associated with higher cerebrospinal HIV-1 viral loads compared to other HIV-1-associated meningitides
Source: PLoS One. 2018 Feb 2;13(2):e0192060. doi: 10.1371/journal.pone.0192060 (PMC5796705; doi:10.1371/journal.pone.0192060)
Supplement: S2 Table — (DOCX) [file pone.0192060.s002.docx]

**S2 table. Analysis of correlation between viral loads with CD4 counts and markers of inflammation (CSF lymphocytes, proteins, and glucose)**

| **HIV Viral loads** | **Plasma** | | | | **Cerebrospinal Fluid** | | | |  |
| --- | --- | --- | --- | --- | --- | --- | --- | --- | --- |
|  | **CD4+** | **Lymphocytes** | **Protein** | **Glucose** | **CD4+** | **Lymphocytes** | **Protein** | **Glucose** |  |
| **All Patients** | **Spearman’s R** | -0.263 | 0.253 | 0.264 | NS | -0.290 | 0.406 | 0.307 | 0.204 |
|  | **P-value** | 0.0117 | 0.018 | 0.0114 | NS | 0.0061 | 0.002 | 0.0039 | 0.0409 |
| **TBM** | **Spearman’s R** | NS | NS | NS | NS | NS | 0.518 | NS | NS |
|  | **P-value** | NS | NS | NS | NS | NS | 0.0240 | NS | NS |
| **Non- TBM** | **Spearman’s R** | NS | NS | NS | NS | NS | 0.410 | NS | NS |
|  | **P-value** | NS | NS | NS | NS | NS | 0.0292 | NS | NS |

NS; not significant
