# Supplementary material for: Tuberculous meningitis is associated with higher cerebrospinal HIV-1 viral loads compared to other HIV-1-associated meningitides
Source: PLoS One. 2018 Feb 2;13(2):e0192060. doi: 10.1371/journal.pone.0192060 (PMC5796705; doi:10.1371/journal.pone.0192060)
Supplement: S3 Table — (DOCX) [file pone.0192060.s003.docx]

**S3 table**. **The predicted odds of having TB meningitis with variation in levels of biomarkers that were significantly different between TBM and non-TBM groups.**

| **Variable** | **Odds ratio**  **(Standard Error)** | **95% confidence intervals** | **P value** |
| --- | --- | --- | --- |
| IL-1β (pg/ml) | 1.053 (0.057) | 0.948-1.170 | 0.332 |
| IL-17 (pg/ml) | 1.034 (0.033) | 0.971-1.102 | 0.301 |
| G-CSF (pg/ml) | 1.001 (0.002) | 0.997-1.005 | 0.584 |
| PDGF-bb (pg/ml) | 1.001 (0.001) | 0.999-1.002 | 0.462 |
| Cathelicidin (ng/ml) | 1.376 (0.227) | 0.996-1.900 | 0.053 |

The predictive ability of CSF IL-1β, IL-17, PDGF-bb, G-CSF and cathelicidin on TBM were predicted in a multivariable logistic regression model.

P values <0.05 were considered significant.
